# Speed-Related Energy Flow and Joint Function Change During Human Walking

**DOI:** 10.3389/fbioe.2021.666428

**Published:** 2021-05-31

**Authors:** Zheqi Hu, Lei Ren, Dan Hu, Yilei Gao, Guowu Wei, Zhihui Qian, Kunyang Wang

**Affiliations:** ^1^Key Laboratory of Bionic Engineering, Ministry of Education, Jilin University, Changchun, China; ^2^School of Mechanical, Aerospace and Civil Engineering, University of Manchester, Manchester, United Kingdom; ^3^School of Mechanical, Aerospace and Automotive Engineering, Coventry University, Coventry, United Kingdom; ^4^School of Science, Engineering and Environment, University of Salford, Salford, United Kingdom

**Keywords:** human walking, biomechanics, speed, energy flow, joint function

## Abstract

During human walking, mechanical energy transfers between segments *via* joints. Joint mechanics of the human body are coordinated with each other to adapt to speed change. The aim of this study is to analyze the functional behaviors of major joints during walking, and how joints and segments alter walking speed during different periods (collision, rebound, preload, and push-off) of stance phase. In this study, gait experiment was performed with three different self-selected speeds. Mechanical works of joints and segments were determined with collected data. Joint function indices were calculated based on net joint work. The results show that the primary functional behaviors of joints would not change with altering walking speed, but the function indices might be changed slightly (e.g., strut functions decrease with increasing walking speed). Waist acts as strut during stance phase and contributes to keep stability during collision when walking faster. Knee of stance leg does not contribute to altering walking speed. Hip and ankle absorb more mechanical energy to buffer the strike during collision with increasing walking speed. What is more, hip and ankle generate more energy during push-off with greater motion to push distal segments forward with increasing walking speed. Ankle also produces more mechanical energy during push-off to compensate the increased heel-strike collision of contralateral leg during faster walking. Thus, human may utilize the cooperation of hip and ankle during collision and push-off to alter walking speed. These findings indicate that speed change in walking leads to fundamental changes to joint mechanics.

## Introduction

Human walking is one of the most significant activities with high efficiency and low metabolic cost in daily living, benefiting from periodic energy generation and absorption (Gordon et al., 1980) which is performed by muscle contractions and soft tissue deformations. Muscle mechanical work (or power) is widely used to compare estimates of the work associated with walking (Cavagna and Kaneko, 1977; Donelan et al., 2002), analyze energy transfer through body segments *via* joints (Caldwell and Forrester, 1992), and evaluate locomotor efficiency (Winter, 1979). To maintain the walking speed, muscles would compensate mechanical energy dissipation with producing positive mechanical work (Kuo et al., 2005). In the literature, the mechanical work is widely analyzed at joint and segment level to present net contributions from muscles, tendons, and other tissues (Zelik et al., 2015).

Joint work changes in the human body are correctly identified for variable speed tasks (Lugade et al., 2014). Different joints may contribute differently to walking (Lee et al., 2008) and joint parameters have strong influences in altering walking speed. According to the conclusion from Farris and Sawicki (2012), the main burst of positive work is performed by ankle at the end of stance phase, which could be determined as push-off (Zelik and Kuo, 2010). Ankle push-off mainly contributes to COM acceleration with increasing speed and kinetic energy of trailing leg (Zelik and Adamczyk, 2016). Sun et al. (2018) suggested that increased sagittal ankle moment is the cause of increased walking speed. Okita et al. (2014) presented that to increase walking speed, human would rely on bilateral hip, ankle, and contralateral knee to generate additional power. With increasing locomotion speeds, the positive work produced by ankle during stance phase, negative work absorbed by knee during swing phase, and positive work produced by hip tend to increase (Ebrahimi et al., 2017; Jin and Hahn, 2018). However, most of them considered the stance phase as a whole, and few were focused on work changes under more precise time period. A more detailed investigation is needed to understand how the mechanical patterns in the joints and segments are coordinated among different stance phases with response to speed changes. Generally, the stance phase of walking gait can be defined based on fluctuating regions of positive and negative individual limb COM power as four different periods: collision, rebound, preload, and push-off (Zelik and Kuo, 2010). This study will try to answer how mechanical energy transfer between segments *via* joints during four different periods of stance phase with changed walking speed.

Joints make different functional contributions to achieve demanded movements, and may reduce energy exchange that could optimize walking economy when the speed changes. It requires the elastic potential properties of the musculotendon system to periodically absorb and generate energy in the stance phase (Cavagna, 1977; Kuitunen et al., 2002; Kuhman and Hurt, 2019). Dickinson (2000) described four basic functional behaviors perform (strut, spring, motor, and damper) based on the mechanical work. Generally, transmitting the forces by muscles during locomotion could be considered as strut, storing, and releasing energy as spring, generating positive power as motor, and absorbing energy as damper. The stance leg acts as strut during walking to reduce total work production in the human body (Cavagna et al., 1976). The lower limb joints serve different functional roles when the walking speed changes, e.g., motor-like function of the ankle and hip would be amplified with increasing speed (Qiao and Jindrich, 2016). However, it is unclear whether the functional behaviors would change during different periods of stance phase (collision, rebound, preload, and push-off) or not.

In this paper, we aim to investigate the joint level mechanics and functional behaviors interaction during walking to further understand the energy flow and joint function when the speeds change. We provide a separate analysis of stance phase joint power and work, providing a more detailed study about joint function of the human body in four different phases of walking across a range of speeds (slow, normal, and fast). A 3D motion capture system integrated with a force plate array was used to measure the kinematic and kinetic data. The mechanical power and work of the joints and segments were calculated based on inverse dynamic analysis. The function indices were characterized from the joint moments and joint work. Statistical analysis has been conducted to evaluate the difference of joint and segment work among altered walking speed, as well as the change of functional behaviors in different phases. Besides, we hypothesized that ankle contributes the most during push-off to alter walking speed. This study would advance the understanding of speed-related joint level mechanics and functional interactions in the human body during walking, which could benefit rehabilitation engineering and the bionic designs of assistive devices such as exoskeleton.

## Materials and Methods

### Gait Measurement

Six healthy adults with no previous medical history of bone or joint injury (*N* = 6, all males; age 26.67 ± 2.69 years; weight 84.25 ± 15.04 kg; height 1.76 ± 0.07 m; mean ± SD) participated in this study. These subjects were previously provided written informed consent before participation and all the experiments were approved by the ethical committee of the university. The whole procedure was in accordance to the World Medical Association *Declaration of Helsinki*. They were asked to walk on a 10-meter-long walkway under three different self-selected speeds: fast (1.82 ± 0.36 m/s), normal (1.51 ± 0.32 m/s), and slow (1.25 ± 0.27 m/s). The walking speed was defined as the stride length divided by time, where the stride length was the displacement of the foot origin from one heel-strike to the next heel-strike. Each speed was measured 10 times to ensure that representative walking data were recorded and used in all the analysis. Kinematic data was collected at 200 Hz using a six-infrared camera motion capture system (Vantage Normal V8, Vicon, United Kingdom), and ground reaction force/moment data were recorded at 1,000 Hz by using a three-force plate array (Type 9281E, Kistler, Switzerland).

The human body was divided into 13 rigid segments (head, torso, pelvis, upper arms, forearms, thighs, shanks, and feet). A group of specially designed thermoplastic plates (Ren et al., 2005) were attached to the segments, each with a cluster of four reflective markers. The head marker cluster was hold by a helmet. The plastic plate holding the pelvis marker cluster has been firmly fixed by an elastic hip belt. Plastic plates and the helmet reduce the relative movement between the markers on a segment, thereby improving the accuracy of the measured data (Angeloni et al., 1993; Garling et al., 2007).

The anatomical landmarks were located from a series of static calibration procedures by using a calibration wand and reflective markers. The calibration markers were then removed before walking tests according to the calibrated anatomical system technique (Cappozzo et al., 1995). The functional approach (Cappozzo, 1984; Gamage and Lasenby, 2002) was used to determine the hip joint center. Other joint centers were defined based on anatomical landmarks.

### Calculation of Joint and Segment Energy

Kinematic and kinetic data were processed by general motion analysis system (GMAS), a MATLAB based package for 3D motion analysis (Ren et al., 2008). With after-processed data, gait parameters such as joint angular velocity and moment are determined. Joint power can be determined with net joint moment (*M*) and joint angular velocity (ɷ) (Winter, 2009) as *P*_*j*_ = *M* ⋅ɷ(1). Segmental power can be described as the sum of joint translational power (*P*_*t*_) and muscle rotational power (*P*_*r*_) at distal and proximal ends (Kautz et al., 1994; Guo et al., 2003) as *P*_*s*_ = *P*_*t,d*_ + *P*_*r,d*_ + *P*_*t,p*_ + *P*_*r,p*_ (2), where the subscript *t* is joint translational power, *r* is muscle rotational power, *d* is the distal end and *p* is the proximal end of the segment. For example, *P*_*t,d*_ means the translational power of the distal segment. Joint translational power equals the dot product of resultant joint force (*F*_*j*_) and joint translational velocity (*v*) as *P*_*t*_ = *F_*j*_ ⋅ v* (3). Muscle rotational power equals the dot product of net joint moment (*M*) and segmental angular velocity (ɷ*_s_*) as *P*_*r*_ = *M* ⋅ɷ*_s_* (4). What is more, the mechanical work produced by joints and mechanical energy change in segments are calculated with integration of joint and segmental power in selected periods (Equation 5).

(5) $$W=\int_{t_{1}}^{t_{2}}P\,dt$$

For example, the mechanical work produced by the femur was calculated as,

(6) $$\begin{array}{c} W_{f\,e\,m\,u\,r}=\int_{t_{1}}^{t_{2}}\left(P_{t,h\,i\,p}+P_{r,h\,i\,p}+P_{t,k\,n\,e\,e}+P_{r,k\,n\,e\,e}\right)\,dt\\ =\int_{t_{1}}^{t_{2}}(F_{h\,i\,p}\cdot \nu_{h\,i\,p}+M_{h\,i\,p}\cdot \omega_{f\,e\,m\,u\,r}+F_{k\,n\,e\,e}\cdot \nu_{k\,n\,e\,e}\\ \;+M_{k\,n\,e\,e}\cdot \omega_{f\,e\,m\,u\,r})dt \end{array}$$

### Calculation of Joint Function Indices

As described by Qiao and Jindrich (2016) and Lai et al. (2019), joint functional behaviors could be characterized as strut-, spring-, motor-, and damper-like based on the mechanical work. In this study, stance phase is divided into four parts: collision, rebound, preload and push-off. Functional indexing analysis was separately conducted based on the mechanical work produced by joints during four different phases.

The strut index equals the ratio of joint mechanical work over moment impulse to determine the joint stiffness (Equation 6). The strut index is great when high moments occur with little movement and little energy fluctuation.

(7) $$s\,t\,r\,u\,t\,\,i\,n\,d\,e\,x=\max\left(1-\frac{\left(t_{2}-t_{1}\right)\,\int_{t_{1}}^{t_{2}}\left|P_{j\,o\,i\,n\,t}\right|\,dt}{\int_{t_{1}}^{t_{2}}\left|M_{j\,o\,i\,n\,t}\,d\,t\right|},0\right)\times 100\%$$

The spring index involves energy absorption during compression (defined as flexion) and energy return during thrust (defined as extension) (Equation 7). The mechanical energy is considered as potentially involved in spring-like behavior as the minimum of negative work during compression and positive work during thrust.

(8) $$\begin{array}{c} s\,p\,r\,i\,n\,g\,\,i\,n\,d\,e\,x=\frac{2\cdot \min\left(\left|W_{c\,o\,m\,p\,r\,e\,s\,s\,i\,o\,n}^{-}\right|,\left|W_{t\,h\,r\,u\,s\,t}^{+}\right|\right)}{\left|W_{T\,o\,t\,a\,l}^{+}\right|+\left|W_{T\,o\,t\,a\,l}^{+}\right|}\\ \times \left(100\%-s\,t\,r\,u\,t\,\,i\,n\,d\,e\,x\right) \end{array}$$

The motor index describes positive work that is not performed *via* spring-like behavior during different phases (Equation 8).

(9) $$\begin{array}{c} m\,o\,t\,o\,r\,\,i\,n\,d\,e\,x=\frac{\left|W_{T\,o\,t\,a\,l}^{+}\right|-\min\left(\left|W_{c\,o\,m\,p\,r\,e\,s\,s\,i\,o\,n}^{-}\right|,\left|W_{t\,h\,r\,u\,s\,t}^{+}\right|\right)}{\left|W_{T\,o\,t\,a\,l}^{-}\right|+\left|W_{T\,o\,t\,a\,l}^{+}\right|}\\ \times \left(100\%-s\,t\,r\,u\,t\,\,i\,n\,d\,e\,x\right) \end{array}$$

The damper index is calculated to measure negative work that is not stored for spring-like behavior (Equation 9).

(10) $$\begin{array}{c} d\,a\,m\,p\,e\,r\,\,i\,n\,d\,e\,x=\frac{\left|W_{T\,o\,t\,a\,l}^{-}\right|-\min\left(\left|W_{c\,o\,m\,p\,r\,e\,s\,s\,i\,o\,n}^{-}\right|,\left|W_{t\,h\,r\,u\,s\,t}^{+}\right|\right)}{\left|W_{T\,o\,t\,a\,l}^{-}\right|+\left|W_{T\,o\,t\,a\,l}^{+}\right|}\\ \times \left(100\%-s\,t\,r\,u\,t\,\,i\,n\,d\,e\,x\right) \end{array}$$

### Statistical Analysis

The statistical analysis was performed to evaluate whether joint/segmental mechanical work and functional behaviors change with different speeds from slow to fast walk using SPSS 20.0 software (IBM, Armonk, NY, United States). For each condition, means and standard errors of joint and segmental work as well as joint functional indices in four different phases were calculated across all subjects and trials. They were then analyzed separately by using the analysis of variance (ANOVA) with repeated measurements based on a linear mixed model approach considering intra- and inter-subject variability (random effects: subjects and trials; fixed effects: walking speed; significance level *p* = 0.05). For *post hoc* processing, we used Fisher’s least significant difference (LSD) multiple comparison based on the least-squared means to compare speed conditions with each other in order to investigate which walking speed exacted a significant change in joint/segmental work and joint functional behaviors.

## Results

### Joint Power and Work

**FIGURE 1:**
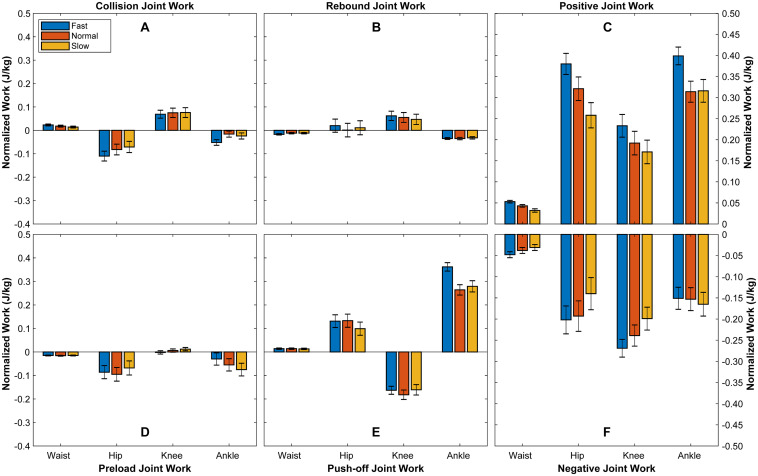
Mechanical works of joints during stance phase. It illustrates the mechanical work produced by each joint during collision **(A)**, rebound **(B)**, preload **(D)**, and push-off **(E)**, as well as the positive **(C)** and negative **(F)** mechanical work produced by each joint during the whole stance phase. Bars depict mean; *N* = 6; error bars, SD.

The results from Table 1 and Figure 1 present how the mechanical work produced by joints change with walking speed. The positive work produced by waist during collision increases approximately 64% from slow to fast walk. The negative work from waist during rebound increases 42% from slow to fast walk. However, the mechanical works produced by waist during preload and push-off do not show significant relevance to walking speed. During the whole stance phase, the positive work from waist increases 66% and negative work absorbed by waist increases 55% from slow to fast walk. The negative work absorbed by hip during collision increases 55% and the positive work during push-off increases 30% from slow to fast walk. During the whole stance phase, the positive work produced by hip increases 47% from slow to fast walk. However, the mechanical works during rebound and preload do not show significant relevance to walking speed. As for the mechanical work produced by knee during stance phase, the positive work increases 36% from slow to fast walking speed. The positive work during preload decreases 50% from slow to normal walking, but knee absorbs negative work under fast walking speed. The negative work absorbed by ankle increases 117% during collision from slow to fast walk but decreases 60% during preload. The positive work produced by ankle during push-off increases 30% from slow to fast walk. In addition, the positive work produced by ankle increases 26% during the whole stance phase from slow to fast walk.

**TABLE 1:** Mechanical work produced by joints during different phases.

Joint	Speed	Collision work	Rebound work	Preload work	Push-off work	Average power	Positive work	Negative work
Waist	Fast	0.023 ± 0.004^a^	−0.017 ± 0.003^a^	−0.015 ± 0.002^a^	0.014 ± 0.003^a^	0.012 ± 0.011^a^	0.053 ± 0.003^a^	−0.048 ± 0.007^a^
	Normal	0.018 ± 0.004^b^	−0.012 ± 0.003^b^	−0.016 ± 0.002^a^	0.014 ± 0.003^a^	0.008 ± 0.011^a^	0.043 ± 0.003^b^	−0.038 ± 0.007^b^
	Slow	0.014 ± 0.004^b^	−0.012 ± 0.003^b^	−0.015 ± 0.002^a^	0.013 ± 0.003^a^	0.003 ± 0.110^a^	0.032 ± 0.004^c^	−0.031 ± 0.007^c^
Hip	Fast	−0.110 ± 0.021^a^	0.020 ± 0.028^a^	−0.086 ± 0.028^a^	0.131 ± 0.027^a^	0.289 ± 0.084^a^	0.380 ± 0.025^a^	−0.202 ± 0.033^a^
	Normal	−0.082 ± 0.023^a,b^	0.001 ± 0.029^a^	−0.095 ± 0.029^a^	0.133 ± 0.028^a^	0.214 ± 0.092^a^	0.321 ± 0.028^b^	−0.193 ± 0.036^a^
	Slow	−0.071 ± 0.024^b^	0.011 ± 0.030^a^	−0.068 ± 0.030^a^	0.099 ± 0.028^b^	0.192 ± 0.098^a^	0.258 ± 0.030^c^	−0.140 ± 0.038^a^
Knee	Fast	0.069 ± 0.017^a^	0.062 ± 0.020^a^	−0.002 ± 0.007^a^	−0.163 ± 0.017^a^	−0.074 ± 0.041^a^	0.233 ± 0.027^a^	−0.269 ± 0.021^a^
	Normal	0.075 ± 0.020^a^	0.055 ± 0.021^a^	0.006 ± 0.007^a,b^	−0.182 ± 0.020^a^	−0.067 ± 0.046^a^	0.192 ± 0.028^b^	−0.239 ± 0.025^a^
	Slow	0.076 ± 0.021^a^	0.047 ± 0.022^a^	0.012 ± 0.007^b^	−0.161 ± 0.022^a^	−0.03 ± 0.050^a^	0.171 ± 0.028^b^	−0.199 ± 0.027^a^
Ankle	Fast	−0.052 ± 0.012^a^	−0.035 ± 0.004^a^	−0.030 ± 0.026^a^	0.362 ± 0.018^a^	0.445 ± 0.070^a^	0.399 ± 0.021^a^	−0.151 ± 0.026^a^
	Normal	−0.026 ± 0.013^b^	−0.035 ± 0.005^a^	−0.055 ± 0.026^a,b^	0.264 ± 0.022^b^	0.272 ± 0.073^b^	0.314 ± 0.025^b^	−0.153 ± 0.027^a^
	Slow	−0.024 ± 0.013^b^	−0.032 ± 0.005^a^	−0.075 ± 0.027^b^	0.279 ± 0.024^b^	0.224 ± 0.074^b^	0.316 ± 0.027^b^	−0.165 ± 0.028^a^

*Data are mean ± SD. for all the trials across all the subjects. Different letters mean that the variable in a column differs significantly with each other (*p* < 0.05). This table contains the mechanical work produced by each joint during collision, rebound, preload, and push-off. The average power presents the average value of joint power during whole stance phase. The positive and negative work present the positive mechanical work and negative mechanical work produced by each joint during whole stance phase.*

### Segmental Power and Work

**FIGURE 2:**
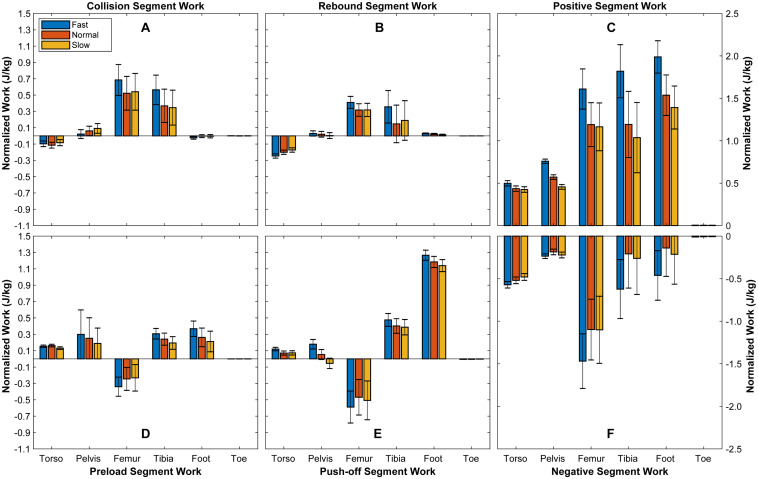
Mechanical energy change of segments during stance phase. It demonstrates the mechanical energy change in each segment during collision **(A)**, rebound **(B)**, preload **(D)**, and push-off **(E)**, as well as the positive **(C)** and negative **(F)** mechanical work applied to each segment during the whole stance phase. Bars depict mean; *N* = 6; error bars, SD.

Table 2 and Figure 2 show the changes of segmental mechanical work with changed walking speed. From slow to fast walk, the positive work applied to torso during stance phase increases 17%, and the mechanical energy released by pelvis increases 59% during preload. During slow walk, pelvis absorbs energy during push-off, however, it releases 238% more mechanical energy during push-off under fast walk than normal walk. During stance phase, pelvis releases 66% more energy from slow to fast walk. The positive mechanical work transferred to foot during push-off increases 11% from slow to fast walk.

**TABLE 2:** Mechanical energy change of segments during different phases.

Segment	Speed	Collision work	Rebound work	Preload work	Push-off work	Average power	Positive work	Negative work
Torso	Fast	−0.097 ± 0.035^a^	−0.247 ± 0.026^a^	0.154 ± 0.014^a^	0.120 ± 0.021^a^	−0.119 ± 0.041^a^	0.499 ± 0.031^a^	−0.572 ± 0.038^a^
	Normal	−0.114 ± 0.036^a^	−0.201 ± 0.026^b^	0.162 ± 0.016^a^	0.071 ± 0.025^a^	−0.136 ± 0.044^a^	0.436 ± 0.032^b^	−0.520 ± 0.039^b^
	Slow	−0.083 ± 0.037^a^	−0.173 ± 0.027^b^	0.130 ± 0.017^a^	0.073 ± 0.026^a^	−0.088 ± 0.045^a^	0.425 ± 0.033^b^	−0.480 ± 0.040^b^
Pelvis	Fast	0.022 ± 0.054^a^	0.030 ± 0.032^a^	0.299 ± 0.025^a^	0.179 ± 0.058^a^	0.942 ± 0.107^a^	0.759 ± 0.055^a^	−0.236 ± 0.027^a^
	Normal	0.061 ± 0.057^a^	0.020 ± 0.035^a^	0.251 ± 0.027^b^	0.053 ± 0.061^b^	0.626 ± 0.112^b^	0.572 ± 0.057^b^	−0.186 ± 0.033^a^
	Slow	0.091 ± 0.059^a^	0.004 ± 0.036^a^	0.188 ± 0.028^c^	−0.055 ± 0.063^c^	0.360 ± 0.114^c^	0.456 ± 0.059^c^	−0.222 ± 0.034^a^
Femur	Fast	0.685 ± 0.190^a^	0.410 ± 0.074^a^	−0.340 ± 0.117^a^	−0.589 ± 0.195^a^	0.158 ± 0.214^a^	1.610 ± 0.237^a^	−1.469 ± 0.321^a^
	Normal	0.522 ± 0.207^a^	0.317 ± 0.077^a^	−0.245 ± 0.140^a^	−0.469 ± 0.217^a^	0.145 ± 0.252^a^	1.190 ± 0.259^a^	−1.098 ± 0.357^a^
	Slow	0.540 ± 0.225^a^	0.318 ± 0.081^a^	−0.232 ± 0.162^a^	−0.507 ± 0.237^a^	0.110 ± 0.290^a^	1.164 ± 0.282^a^	−1.101 ± 0.394^a^
Tibia	Fast	0.564 ± 0.181^a^	0.356 ± 0.199^a^	0.307 ± 0.064^a^	0.476 ± 0.078^a^	2.004 ± 0.249^a^	1.820 ± 0.313^a^	−0.623 ± 0.346^a^
	Normal	0.368 ± 0.204^a^	0.147 ± 0.230^a^	0.241 ± 0.074^a^	0.402 ± 0.089^a^	1.677 ± 0.285^a^	1.192 ± 0.390^a^	−0.209 ± 0.402^a^
	Slow	0.346 ± 0.214^a^	0.189 ± 0.243^a^	0.194 ± 0.078^a^	0.387 ± 0.094^a^	1.387 ± 0.301^a^	1.037 ± 0.414^a^	−0.262 ± 0.424^a^
Foot	Fast	−0.024 ± 0.016^a^	0.031 ± 0.005^a^	0.368 ± 0.094^a^	1.266 ± 0.062^a^	2.567 ± 0.293^a^	1.988 ± 0.191^a^	−0.462 ± 0.291^a^
	Normal	−0.003 ± 0.018^a^	0.024 ± 0.007^a^	0.262 ± 0.114^a^	1.184 ± 0.068^a,b^	2.283 ± 0.320^a,b^	1.537 ± 0.239^a^	−0.140 ± 0.333^a^
	Slow	−0.004 ± 0.020^a^	0.014 ± 0.007^a^	0.212 ± 0.126^a^	1.139 ± 0.073^b^	1.828 ± 0.330^b^	1.392 ± 0.253^a^	−0.214 ± 0.351^a^

*Data are mean ± SD. for all the trials across all the subjects. Different letters mean that the variable in a column differs significantly with each other (*p* < 0.05). This table contains the mechanical energy change of each segment during collision, rebound, preload, and push-off. The average power presents the average value of segment power during whole stance phase. The positive and negative work present the positive mechanical work and negative mechanical work applied to each segment during whole stance phase.*

### Joint Function Indices

**FIGURE 3:**
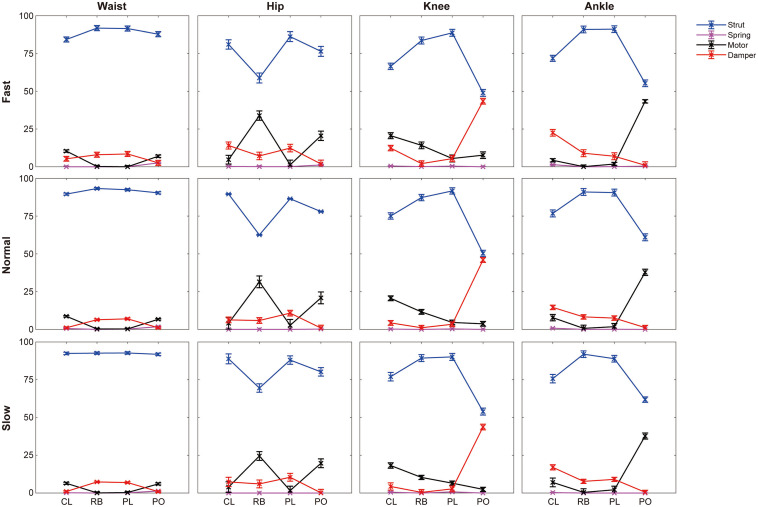
Functional behaviors of joints during different phases across three walking speeds. *N* = 6; mean ± SD. were depicted. CL, collision; RB, rebound; PL, preload; PO, push-off.

Here, the joint indices under normal walking were used to demonstrate the change of joint functional behaviors during stance phases, while the variation trend of joint indices under the other two speeds are similar (Figure 3 and Supplementary Table 1). For the waist, the strut functions during rebound (93.3%) and preload (92.5%) are larger than collision (89.6%) and push-off (90.4%), the motor-like functions during collision (8.7%) and push-off (6.7%) are larger than rebound (0.3%) and preload (0.3%), and the damper-like functions during rebound (6.4%) and preload (7%) are larger than collision (1.1%) and push-off (1.1%). The strut index of hip decreases from collision (89.6%) to rebound (62.6%), then increases during preload (86.5%), and decreases during push-off (78%). The motor-like function of hip increases from collision (4.1%) to rebound (31.5%), then decreases during preload (2.7%), and increases during push-off (20.9%). The damper-like function of hip increases from collision (6.3%) to preload (10.8%) but decreases during push-off (0.9%). For the knee, the strut index increases from collision (75.1%) to preload (91.7%) but decreases during push-off (50.3%), the motor index decreases from collision (20.6%) to push-off (3.7), and the damper index increases from preload (3.4%) to push-off (46%). Regarding the ankle, the strut index increases from collision (76.8%) to preload (90.6%) but decreases during push-off (61%), the motor index decreases from collision (7.8%) to preload (1.8%) but increases during push-off (37.8%), and the damper index decreases from collision (14.6%) to push-off (1.2%).

**FIGURE 4:**
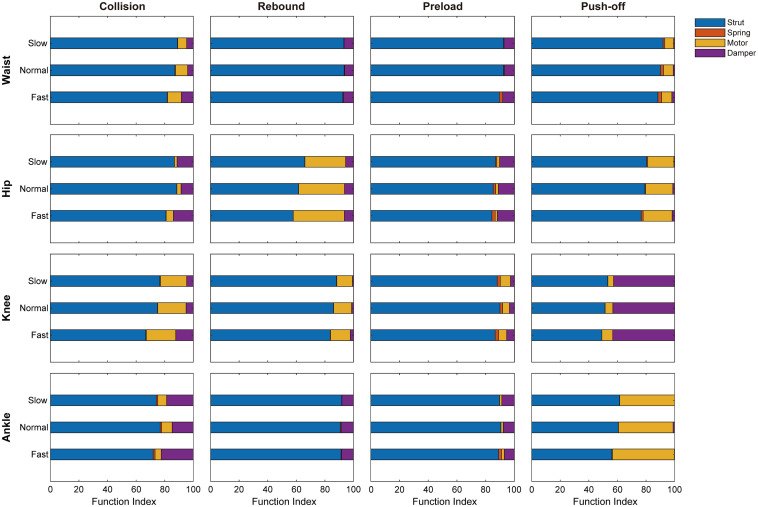
The functional indices of joints during different phases across three walking speeds. *N* = 6; means were depicted.

Moreover, Figure 4 and Table 3 depict the relevance of joint indices to walking speed. Increasing walking speed involves increased waist’s motor-like function during collision and damper-like function during push-off, but decreased strut-like function. During collision, motor-like function of hip is amplified with increased walking speed. Also, walking speed boost involves growing motor-like function of hip but decreasing strut-like function during push-off. With walking speed being increased, the damper-like function of knee during collision and preload increases, but the strut-like function decreases during all the subphases except for push-off. During collision, damper-like function of ankle increases but motor-like function decreases with increasing walking. Contrarily, during push-off, damper-like function decreases but motor-like function increases.

**TABLE 3:** The functional indices of joints during different phases across three walking speeds.

**Collision**						**Preload**					
	
**Joint**	**Speed**	**Strut**	**Spring**	**Motor**	**Damper**	**Joint**	**Speed**	**Strut**	**Spring**	**Motor**	**Damper**
Waist	Fast	81.7 ± 2.7^a^	0.2 ± 0.2^a^	9.7 ± 1.0^a^	8.4 ± 3.0^a^	Waist	Fast	89.5 ± 1.9^a^	2.3 ± 0.1^a^	0.0 ± 0.1^a^	8.2 ± 1.0^a^
	Normal	87.1 ± 3.4^a^	0.4 ± 0.3^a^	8.5 ± 1.4^a^	4.0 ± 3.5^a^		Normal	92.4 ± 2.2^a^	0.2 ± 0.1^a^	0.3 ± 0.1^a^	7.1 ± 1.0^a^
	Slow	88.9 ± 3.5^a^	0.2 ± 0.2^a^	6.2 ± 1.4^a,b^	4.7 ± 3.6^a^		Slow	92.4 ± 2.4^a^	0.1 ± 0.2^a^	0.2 ± 0.1^a^	7.3 ± 1.0^a^
Hip	Fast	80.8 ± 3.0^a^	0.2 ± 0.1^a^	5.0 ± 0.7^a^	14.0 ± 2.9^a^	Hip	Fast	84.3 ± 2.6^a^	2.3 ± 0.0^a^	1.5 ± 0.8^a^	11.9 ± 1.6^a^
	Normal	88.4 ± 3.7^a^	0.1 ± 0.2^a^	2.9 ± 0.9^b^	8.6 ± 3.5^a^		Normal	85.2 ± 3.0^a^	1.7 ± 0.0^a^	1.9 ± 0.9^a^	11.2 ± 1.8^a^
	Slow	86.8 ± 4.6^a^	0.0 ± 0.2^a^	1.8 ± 1.1^b^	11.4 ± 4.3^a^		Slow	86.8 ± 3.1^a^	0.9+0.0^a^	1.9 ± 0.9^a^	10.4 ± 1.9^a^
Knee	Fast	66.7 ± 2.9^a^	0.4 ± 0.3^a^	20.6 ± 2.7^a^	12.3 ± 2.3^a^	Knee	Fast	86.6 ± 2.3^a^	2.4 ± 0.2^a^	5.6 ± 1.1^a^	5.4 ± 0.9^a^
	Normal	74.9 ± 3.5^b^	0.2 ± 0.2^a^	19.8 ± 3.4^a^	5.1 ± 2.8^b^		Normal	89.9 ± 2.4^b^	1.8 ± 0.3^a^	4.8 ± 1.2^a^	3.5 ± 1.0^b^
	Slow	76.5 ± 4.4^b^	0.5 ± 0.4^a^	18.3 ± 4.5^a^	4.7 ± 3.8^b^		Slow	88 ± 2.4^a,b^	2.1 ± 0.3^a^	7.1 ± 1.3^a^	2.8 ± 1.1^b^
Ankle	Fast	71.9 ± 2.4^a^	1.3 ± 0.3^a^	4.3 ± 1.1^a^	22.5 ± 3.2^a^	Ankle	Fast	89.0 ± 2.3^a^	2.0 ± 0.0^a^	1.9 ± 0.9^a^	7.1 ± 2.9^a^
	Normal	76.8 ± 3.1^a^	1.0 ± 0.3^a^	7.4 ± 1.2^b^	14.8 ± 3.8^b^		Normal	90.5 ± 2.7^a^	0.1 ± 0.0^a^	1.6 ± 0.9^a^	7.8 ± 3^a,b^
	Slow	74.1 ± 4.1^a^	1.0 ± 0.3^a^	6.2 ± 1.4^a,b^	18.7 ± 4.8^a,b^		Slow	89.5 ± 2.8^a^	0.1 ± 0.0^a^	1.6 ± 0.9^a^	8.8 ± 0.3^b^

**Rebound**						**Push-off**					
	
**Joint**	**Speed**	**Strut**	**Spring**	**Motor**	**Damper**	**Joint**	**Speed**	**Strut**	**Spring**	**Motor**	**Damper**

Waist	Fast	92.5 ± 1.0^a^	0.1 ± 0.0^a^	0.3 ± 0.2^a^	7.1 ± 1.1^a^	Waist	Fast	88.3 ± 0.8^a^	2.5 ± 0.9^a^	7.2 ± 1.2^a^	2.0 ± 0.4^a^
	Normal	93.4 ± 1.1^a^	0.1 ± 0.0^a^	0.3 ± 0.2^a^	6.2 ± 1.1^a^		Normal	90.1 ± 0.9^b^	2.1 ± 1.0^a^	6.8 ± 1.2^a^	1.0 ± 0.5^b^
	Slow	93.2 ± 1.1^a^	0.0 ± 0.0^a^	0.2 ± 0.2^a^	6.6 ± 1.2^a^		Slow	91.9 ± 0.9^c^	1.2 ± 1.0^a^	6.2 ± 1.3^a^	0.7 ± 0.5^c^
Hip	Fast	57.8 ± 6.6^a^	0.0 ± 0.0^a^	35.8 ± 6.8^a^	6.4 ± 3.3^a^	Hip	Fast	76.60 ± 1.50^a^	1.4 ± 1.2^a^	20.2 ± 0.23^a^	1.8 ± 0.8^a^
	Normal	61.5 ± 6.9^a^	0.1 ± 0.0^a^	32.1 ± 7.3^a^	6.3 ± 3.7^a^		Normal	79.20 ± 1.70^a^	0.5 ± 1.2^a^	19.0 ± 2.4^a^	1.3 ± 0.9^a^
	Slow	65.9 ± 7.1^a^	0.1 ± 0.0^a^	28.5 ± 7.6^a^	5.5 ± 3.9^a^		Slow	80.40 ± 0.18^a^	0.8 ± 1.2^a^	18.3 ± 2.5^a^	0.5 ± 0.9^a^
Knee	Fast	83.7 ± 3.1^a^	0.2 ± 0.0^a^	13.8 ± 3.2^a^	2.3 ± 0.8^a^	Knee	Fast	49.1 ± 3.4^a^	0.0 ± 0.0^a^	7.6 ± 2.1^a^	43.3 ± 3.1^a^
	Normal	86.0 ± 3.2^a^	0.1 ± 0.1^a^	12.5 ± 3.3^a^	1.4 ± 0.8^a^		Normal	51.3 ± 3.7^a,b^	0.1 ± 0.0^a^	5.4 ± 2.2^a,b^	43.2 ± 3.6^a^
	Slow	88.1 ± 3.2^b^	0.2 ± 0.0^a^	10.9 ± 3.3^a^	0.8 ± 0.9^a^		Slow	53.2 ± 3.8^b^	0.1 ± 0.0^a^	4.0 ± 2.3^b^	42.7 ± 3.8^a^
Ankle	Fast	91.30 ± 1.20^a^	0.0 ± 0.0^a^	0.3 ± 0.2^a^	8.40 ± 0.13^a^	Ankle	Fast	56.1 ± 2.6^a^	0.4 ± 0.0^a^	43.2 ± 2.7^a^	0.3 ± 0.3^a^
	Normal	90.70 ± 0.13^a^	0.1 ± 0.0^a^	0.5 ± 0.2^a^	8.70 ± 1.40^a^		Normal	60.6 ± 2.9^a^	0.1 ± 0.0^a^	38.2 ± 3.0^a^	1.1 ± 0.4^a^
	Slow	91.70 ± 1.40^a^	0.0 ± 0.0^a^	0.2 ± 0.3^a^	8.10 ± 1.40^a^		Slow	61.5 ± 3.1^a^	0.1 ± 0.0^a^	38.4 ± 3.1^a^	0.0 ± 0.0^a^

*Data are mean ± SD. for all the trials across all the subjects. Different letters mean that the variable in a column differs significantly with each other (*p* < 0.05).*

## Discussion

### The Primary Functional Behaviors of Joints Would Not Change With Walking Speed

Qiao and Jindrich (2016) presented that primary functional behavior of joint could be determined with the most proportional function index. In this study, the primary functional behaviors of joints are strut-like function during different phases, which appear some differences with previously reported by Qiao and Jindrich (2016). They indicated that hip acted as motor and ankle acted as spring during human walking. These discrepancies are mainly caused from different calculating time periods and terms. We performed separate calculation during four subphases of stance phase and included the energy from all three degree of freedoms (but only flexion-extension in the previous study).

Besides, slight changes occur in function indices with different walking speed. Kuhman and Hurt (2019) suggested that joints’ functional behaviors during walking, especially for knee and ankle, were different under changed walking speed. The results in this study show that, strut function of knee during collision and push-off decreases with growing walking speed (seen in Table 3). Also, increasing walking speed leads to increased damper index of knee but decreased damper index of ankle during preload. However, strut indices are still the most proportional among them. Thus, during changed walking speed, there is no substantial change in functional behaviors of different phases.

### Waist and Knee Did Not Involve Altering Walking Speed but Waist Provided Stability During Collision With Increasing Speed

During the rebound, preload and push-off, the strut indices of waist are larger than 88%. The mechanical energy produced by waist is much less than that of the other joints during whole stance phase. Accordingly, it can be concluded that waist mainly acts as strut during stance phase. Moreover, mechanical energy generation and dissipation of waist mainly occur during collision. Previous researches (Donelan et al., 2004; Schulz et al., 2005) presented that waist played an important role in active lateral stabilization. Therefore, mechanical energy generation and dissipation of waist may be utilized to keep stability during collision. The negative mechanical works from waist during collision is enlarged with increasing walking speed, suggesting that the muscle group surrounding waist absorbs more mechanical energy to keep stability during collision under faster walking. As the inputted energy from waist to pelvis decreases with increasing walking speed, meanwhile the outputted energy from torso to waist does not show significant change with speed, the increased mechanical energy absorbed by waist is mainly from pelvis during collision. It suggests that pelvis helps to keep stability with absorbing less energy during collision when walking faster.

During different speed walking, the strut-like function of waist during push-off decreases with increasing walking speed, same as the strut-like function of knee during collision and push-off. Jeon et al. (2020) revealed that decreased leg stiffness was associated with greater displacement of leg movement. Therefore, the change of waist and knee strut function may lead greater leg movement during collision and push-off when walking speed is raised up.

However, the mechanical work produced by waist and knee do not show significantly relevance to altering walking speed. According to Qiao and Jindrich (2016), joint work’s changes are mainly associated with joint angular displacement changes, not moment. Therefore, angular displacement of waist and knee may not change significantly with walking speed. Greater leg movement would be associated with hip and ankle. This is supported by the finding from Okita et al. (2014) that altering walking speed relies on contralateral knee. Jin and Hahn (2018) also suggested that more negative work would be absorbed by knee during swing phase with increasing locomotion speed. Therefore, knee of stance leg does not contribute to altering walking speed.

### Hip Transformed More Energy From Damper at Collision to Motor at Push-Off With Increasing Speed Due to Translation Work Change of Pelvis and Femur

Regarding hip joint functional behaviors, we observe that only motor-like function during collision is enhanced with increasing walking speed. The results of hip power reveal that the mechanical work produced by hip during collision and push-off increases significantly under faster walking speed. A similar result was presented by Arnold et al. (2013) that the greatest positive work increases occurred at hip and ankle. Another finding in this study is that during collision, the mechanical energy absorbed by hip increases 55% from slow to fast walk. During collision, the human body absorbs negative work to buffer the stride. Hip contributes more to buffering the stride under increasing walking speed. As human walking is not all hard work (Zelik and Kuo, 2010), the contributions of hip may be mainly from muscle group surrounding hip and soft tissue between pelvis and thigh. Besides, the mechanical energy generated by hip increases 30% during push-off from slow to fast walk, suggesting that the muscle group surrounding hip produces more mechanical energy to push pelvis forward by increasing angular motion. This is supported by the inference above and the conclusions of previous researches (Ogihara et al., 2010; Yang et al., 2019; Okudaira et al., 2020).

During segmental mechanical work calculation, the translational and rotational work at both ends of segments are determined (Supplementary Table 2). We observe that the mechanical energy absorbed from hip to pelvis and femur due to translational work during collision grows 26% from slow to fast walk. Meanwhile, the mechanical energy released to hip from pelvis and thigh due to translational work during push-off increases 29% from slow to fast walk. In addition to the greater angular motion, hip transfers more energy from joint reaction forces during collision and push-off when walking faster. It could also be observed that more mechanical energy is inputted to pelvis from hip under faster walking during push-off. Therefore, hip transfers more energy to pelvis for forward propulsion during push-off.

In conclusion, hip works as damper during collision but as motor during push-off, and transfers more energy with increasing speed due to the translational work change of pelvis and femur. The increased angular motion and mechanical energy of hip during push-off involve faster walking speed.

### Ankle Contributes the Most During Push-Off to Push Shank Faster During Walking

From the previous researches (Cofré et al., 2011; Williams and Schache, 2016; Browne and Franz, 2018; Uematsu et al., 2018), ankle produces positive work during stance phase, which could be regarded as motor-like function. However, some studies regarded ankle as principle spring during walking (Lee et al., 2008; Qiao and Jindrich, 2016; Kuhman and Hurt, 2019). In this study, according to the functional behaviors and produced mechanical work of ankle during different phases, we show that ankle is dissipating mechanical energy during the first three phases (collision, rebound, and preload) and releasing mechanical energy during push-off. Moreover, the released mechanical work is larger than the sum of absorbed mechanical work. The results indicate that ankle works as motor to generate mechanical energy during push-off and as spring to store and release energy during the whole stance phase (Figure 1).

Specifically, ankle absorbed 117% more work during collision from slow to fast walk. It reveals that the muscle group surrounding ankle along with soft tissue between shank and foot absorb more mechanical energy during stride with increasing walking speed. As the amplitudes of vertical ground reaction forces are larger in fast walk (Sun et al., 2018), the increased mechanical energy absorbed by muscle group and soft tissue surrounding hip and ankle may be generated from higher ground reaction forces. Ankle absorbs 60% less work during preload from slow to fast walk. During preload elastic energy is stored in the soft tissue, and subsequently released to generate positive external mechanical work (Donelan et al., 2002). Therefore, there are less energy stored during preload and released during push-off with increasing speed, which may be caused by shorter period of preload. During push-off, ankle produces 30% more positive work from slow to fast walk. As the stored energy during preload decreases with increasing speed, more than 30% positive mechanical work is generated by muscle group surrounding ankle. Push-off can compensate and reduce amount of heel-strike collision (Sánchez et al., 2019). Thus, the increased mechanical work produced by ankle during push-off with greater movement may be utilized to push shank forward harder and compensate the increased heel-strike collision of contralateral leg.

The results of calculated segmental work (Table 2 and Figure 2) show that foot contributes to walking with absorbing energy mainly during push-off, supported by previous studies (Safaeepour et al., 2014; Ebrahimi et al., 2017; Welte et al., 2018). Moreover, the mechanical work applied to foot increases during push-off when walking speed being increased. Meanwhile, Hedrick et al. (2019) characterized that the foot and ankle synthesize the force, displacement, and work distal to the shank. Thus, when walking speed being increased, ankle and foot cooperate to push shank faster.

## Conclusion

By calculating and statistically analyzing the joint and segmental work along with the functional behaviors of joints, we found that speed changing during walking is a cooperative work of different joints, especially hip and ankle. Waist mainly works on stabilization during collision under different walking speed. Knee of stance leg does not contribute to altering walking speed. The muscle group and soft tissue surrounding hip and ankle absorb more mechanical energy from higher ground reaction forces during heel-strike. Furthermore, hip and ankle generate more mechanical energy with greater motion during push-off to push distal segments forward with increasing walking speed. Ankle generates more mechanical energy during push-off to compensate the increased heel-strike collision of contralateral leg during faster walking. Overall, the hypothesis provided at the beginning can be confirmed and improved to that both hip and ankle contribute to altering walking speed during collision and push-off.

## Data Availability Statement

The original contributions presented in the study are included in the article/Supplementary Material, further inquiries can be directed to the corresponding author/s.

## Ethics Statement

The studies involving human participants were reviewed and approved by the ethical committee of the university. The patients/participants provided their written informed consent to participate in this study.

## Author Contributions

LR, GW, and ZQ contributed to the concept and design of the research. ZH, DH, YG, and KW contributed to the data collection and processing. ZH, LR, and KW were responsible for the manuscript preparation, discussion, and revision. All the authors involved in finalizing the manuscript.

## Conflict of Interest

The authors declare that the research was conducted in the absence of any commercial or financial relationships that could be construed as a potential conflict of interest.
