# Supplementary material for: Speed-Related Energy Flow and Joint Function Change During Human Walking
Source: Front Bioeng Biotechnol. 2021 May 31;9:666428. doi: 10.3389/fbioe.2021.666428 (PMC8201992; doi:10.3389/fbioe.2021.666428)
Supplement: Supplementary file 1 [file Table_1.DOCX]

***Supplementary Material***

**Supplementary Table S1.** The functional indices of joints under different walking speeds across four stance phases.

| Fast walk | | | | | |
| --- | --- | --- | --- | --- | --- |
| Joint | **Period** | **Strut** | **Spring** | **Motor** | **Damper** |
| Waist | **Collision** | 84.3±1.7^a^ | 0.00±0.40^a^ | 10.4±0.8^a^ | 5.3±1.6^a^ |
|  | **Rebound** | 91.8±1.7^b^ | 0.00±0.40^a^ | 0.2±0.8^b^ | 8.0±1.7^b^ |
|  | **Preload** | 91.5±1.7^b^ | 0.00±0.04^a^ | 0.0±0.8^b^ | 8.5±1.7^b^ |
|  | **Push-off** | 87.8±1.7^c^ | 2.60±0.40^b^ | 7.0±0.8^a^ | 2.6±1.7^c^ |
| Hip | **Collision** | 81.0±3.1^a^ | 0.2±0.4^a^ | 4.7±2.9^a^ | 14.1±2.3^a^ |
|  | **Rebound** | 58.8±3.3^b^ | 0.1±0.4^a^ | 33.9±3.1^b^ | 7.2±2.5^b^ |
|  | **Preload** | 86.2±3.3^a^ | 0.1±0.4^a^ | 1.3±3.1^a^ | 12.4±2.5^a,b^ |
|  | **Push-off** | 76.4±3.3^a,c^ | 1.2±0.4^b^ | 20.5±3.1^c^ | 1.9±2.5^b^ |
| Knee | **Collision** | 66.5±2.1^a^ | 0.5±0.2^a^ | 20.6±2^a^ | 12.4±1.8^a^ |
|  | **Rebound** | 83.6±2.3^b^ | 0.1±0.2^a^ | 14.2±2.2^b^ | 2.1±1.9^b^ |
|  | **Preload** | 88.7±2.3^b^ | 0.4±0.2^a^ | 5.5±2.3^c^ | 5.4±2.0^b^ |
|  | **Push-off** | 48.9±2.3^c^ | 0.0±0.2^a^ | 7.7±2.2^c^ | 43.4±1.9^c^ |
| Ankle | **Collision** | 71.8±2.0^a^ | 1.4±0.3^a^ | 4.3±1.1^a^ | 22.5±2.2^a^ |
|  | **Rebound** | 90.9±2.2^b^ | 0.0±0.3^b^ | 0.1±1.1^b^ | 9.0±2.3^b^ |
|  | **Preload** | 91.1±2.2^b^ | 0.1±0.3^b^ | 1.8±1.1^b^ | 7.0±2.3^b^ |
|  | **Push-off** | 55.3±2.2^c^ | 0.4±0.3^b^ | 43.3±1.1^c^ | 1.0±2.3^c^ |
|  |  |  |  |  |  |
|  |  |  |  |  |  |
| Normal walk | | | | | |
| Joint | **Period** | **Strut** | **Spring** | **Motor** | **Damper** |
| Waist | **Collision** | 89.6±0.7^a^ | 0.6±0.5^a^ | 8.7±0.5^a^ | 1.1±0.6^a^ |
|  | **Rebound** | 93.3±0.7^b^ | 0.0±0.5^a^ | 0.3±0.5^b^ | 6.4±0.6^a^ |
|  | **Preload** | 92.5±0.7^b^ | 0.2±0.5^a^ | 0.3±0.5^b^ | 7.0±0.6^a^ |
|  | **Push-off** | 90.4±0.7^a^ | 1.8±0.5^b^ | 6.7±0.5^c^ | 1.1±0.6^a^ |
| Hip | **Collision** | 89.6±0.3^a^ | 0.0±0.1^a^ | 4.1±3.9^a^ | 6.3±1.9^a^ |
|  | **Rebound** | 62.6±0.3^b^ | 0.0±0.1^a^ | 31.5±3.9^b^ | 5.9±1.9^a^ |
|  | **Preload** | 86.5±0.3^a^ | 0.0±0.1^a^ | 2.7±3.9^a^ | 10.8±1.9^b^ |
|  | **Push-off** | 78.0±0.3^c^ | 0.2±0.1^a^ | 20.9±3.9^c^ | 0.9±1.9^c^ |
| Knee | **Collision** | 75.1±2.1^a^ | 0.2±0.1^a^ | 20.6±1.6^a^ | 4.3±1.6^a^ |
|  | **Rebound** | 87.3±2.1^b^ | 0.0±0.1^a^ | 11.6±1.6^b^ | 1.1±1.6^a^ |
|  | **Preload** | 91.7±2.1^c^ | 0.3±0.1^a^ | 4.6±1.6^c^ | 3.4±1.6^a^ |
|  | **Push-off** | 50.3±2.1^d^ | 0.0±0.1^a^ | 3.7±1.6^c^ | 46±1.6^b^ |
| Ankle | **Collision** | 76.8±2.3^a^ | 0.8±0.1^a^ | 7.8±2.1^a^ | 14.6±1.5^a^ |
|  | **Rebound** | 91.0±2.3^b^ | 0.0±0.1^b^ | 0.7±2.1^b^ | 8.3±1.5^b^ |
|  | **Preload** | 90.6±2.3^b^ | 0.1±0.1^b^ | 1.8±2.1^b^ | 7.5±1.5^b^ |
|  | **Push-off** | 61.0±2.3^c^ | 0.0±0.1^b^ | 37.8±2.1^c^ | 1.2±1.5^c^ |
|  |  |  |  |  |  |
|  |  |  |  |  |  |
| Slow walk | | | | | |
| Joint | **Period** | **Strut** | **Spring** | **Motor** | **Damper** |
| Waist | **Collision** | 92.4±0.8^a^ | 0.2±0.2^a^ | 6.4±0.7^a^ | 1.0±0.5^a^ |
|  | **Rebound** | 92.6±0.8^a^ | 0.0±0.2^a^ | 0.1±0.7^b^ | 7.3±0.5^b^ |
|  | **Preload** | 92.7±0.8^a^ | 0.1±0.2^a^ | 0.3±0.7^b^ | 6.9±0.5^b^ |
|  | **Push-off** | 91.8±0.8^a^ | 1.3±0.2^b^ | 6.0±0.7^a^ | 0.9±0.5^a^ |
| Hip | **Collision** | 88.8±3.3^a^ | 0.0±0.0^a^ | 3.9±3.5^a^ | 7.3±3.1^a^ |
|  | **Rebound** | 69.5±2.8^b^ | 0.0±0.0^a^ | 24.5±2.9^b^ | 6.0±2.6^a^ |
|  | **Preload** | 88.0±2.8^a^ | 0.0±0.0^a^ | 1.6±2.9^a^ | 10.4±2.6^a^ |
|  | **Push-off** | 80.2±2.8^c^ | 0.0±0.0^a^ | 19.7±2.9^b^ | 0.1±2.3^b^ |
| Knee | **Collision** | 77.0±2.8^a^ | 0.5±0.3^a^ | 18.2±1.8^a^ | 4.3±2.4^a^ |
|  | **Rebound** | 89.3±2.3^b^ | 0.0±0.2^a^ | 10.3±1.4^b^ | 0.4±1.9^a^ |
|  | **Preload** | 90.1±2.3^b^ | 0.7±0.2^a^ | 6.5±1.4^c^ | 2.7±1.9^a^ |
|  | **Push-off** | 53.9±2.3^c^ | 0.0±0.2^a^ | 2.4±1.4^d^ | 43.7±1.9^b^ |
| Ankle | **Collision** | 75.7±2.8^a^ | 0.3±0.1^a^ | 7.0±2.9^a^ | 17.0±1.7^a^ |
|  | **Rebound** | 91.9±2.2^b^ | 0.0±0.1^b^ | 0.4±2.4^b^ | 7.7±1.4^b^ |
|  | **Preload** | 88.9±2.2^b^ | 0.0±0.1^b^ | 2.1±2.4^b^ | 9.0±1.4^b^ |
|  | **Push-off** | 61.8±1.8^c^ | 0.0±0.1^b^ | 37.7±2.0^c^ | 0.5±1.3^c^ |
| Data are mean ± s.d. for all the trials across all the subjects. Different letters mean that the variable in a column differs significantly with each other (*p* < 0.05) | | | | | |

**Supplementary Table S2.** Energy flow statistical analysis.

| **Segment** | **Joint** | **Work Type** | **Speed** | **Collision Work** | **Rebound Work** | **Preload Work** | **Push-off Work** |
| --- | --- | --- | --- | --- | --- | --- | --- |
| **Torso** | **Waist** | **Rotational** | **Fast** | -0.007±0.002^a^ | 0.002±0.001^a^ | 0.004±0.001^a^ | -0.007±0.002^a^ |
|  |  |  | **Normal** | -0.007±0.002^a^ | 0.001±0.002^a^ | 0.005±0.001^a^ | -0.005±0.002^a^ |
|  |  |  | **Slow** | -0.006±0.002^a^ | 0.002±0.002^a^ | 0.004±0.001^a^ | -0.007±0.002^a^ |
|  |  | **Translational** | **Fast** | -0.213±0.024^a^ | -0.140±0.024^a^ | 0.215±0.026^a^ | 0.069±0.024^a^ |
|  |  |  | **Normal** | -0.183±0.024^b^ | -0.139±0.024^a^ | 0.231±0.027^a,b^ | 0.011±0.026^b^ |
|  |  |  | **Slow** | -0.169±0.024^b^ | -0.121±0.024^b^ | 0.237±0.027^b^ | -0.032±0.027^c^ |
| **Pelvis** | **Waist** | **Rotational** | **Fast** | 0.015±0.003^a^ | -0.014±0.002^a^ | -0.011±0.002^a^ | 0.007±0.003^a^ |
|  |  |  | **Normal** | 0.011±0.003^b^ | -0.010±0.002^b^ | -0.011±0.002^a^ | 0.008±0.003^a^ |
|  |  |  | **Slow** | 0.006±0.003^c^ | -0.009±0.002^b^ | -0.008±0.002^a^ | 0.005±0.003^a^ |
|  |  | **Translational** | **Fast** | -0.213±0.024^a^ | -0.140±0.024^a^ | 0.215±0.026^a^ | 0.069±0.024^a^ |
|  |  |  | **Normal** | -0.183±0.024^b^ | -0.139±0.024^a^ | 0.231±0.027^a,b^ | 0.011±0.026^b^ |
|  |  |  | **Slow** | -0.169±0.024^b^ | -0.121±0.024^b^ | 0.237±0.027^b^ | -0.032±0.027^c^ |
|  | **Hip** | **Rotational** | **Fast** | 0.045±0.010^a^ | -0.030±0.005^a^ | -0.018±0.009^a^ | -0.000409±0.010000^a^ |
|  |  |  | **Normal** | 0.040±0.010^a^ | -0.035±0.006^a^ | -0.009±0.009^b^ | -0.008000±0.010000^a^ |
|  |  |  | **Slow** | 0.038±0.010^a^ | -0.030±0.006^a^ | -0.018±0.009^b^ | -0.008000±0.010000^a^ |
|  |  | **Translational** | **Fast** | 0.373±0.030^a^ | 0.206±0.040^a^ | -0.132±0.032^a^ | -0.206±0.039^a^ |
|  |  |  | **Normal** | 0.314±0.031^b^ | 0.191±0.040^a^ | -0.164±0.033^b^ | -0.184±0.040^a,b^ |
|  |  |  | **Slow** | 0.296±0.031^b^ | 0.167±0.040^b^ | -0.202±0.033^c^ | -0.160±0.041^b^ |
| **Femur** | **Hip** | **Rotational** | **Fast** | 0.014±0.013^a^ | 0.043±0.033^a^ | -0.091±0.022^a^ | 0.181±0.017^a^ |
|  |  |  | **Normal** | 0.009±0.014^a^ | 0.027±0.034^a^ | -0.099±0.023^a^ | 0.168±0.019^a^ |
|  |  |  | **Slow** | 0.025±0.014^a^ | 0.032±0.035^a^ | -0.082±0.024^a^ | 0.157±0.020^a^ |
|  |  | **Translational** | **Fast** | 0.373±0.030^a^ | 0.206±0.04^a^ | -0.132±0.032^a^ | -0.206±0.039^a^ |
|  |  |  | **Normal** | 0.314±0.031^b^ | 0.191±0.04^a^ | -0.164±0.033^b^ | -0.184±0.040^a,b^ |
|  |  |  | **Slow** | 0.296±0.031^b^ | 0.167±0.04^b^ | -0.202±0.033^c^ | -0.160±0.041^b^ |
|  | **Knee** | **Rotational** | **Fast** | -0.094±0.019^a^ | -0.085±0.033^a^ | 0.037±0.013^a^ | 0.090±0.012^a^ |
|  |  |  | **Normal** | -0.073±0.020^a,b^ | -0.083±0.034^a^ | 0.012±0.015^a^ | 0.089±0.012^a^ |
|  |  |  | **Slow** | -0.054±0.020^b^ | -0.055±0.035^a^ | 0.020±0.016^a^ | 0.083±0.013^a^ |
|  |  | **Translational** | **Fast** | 0.366±0.025^a^ | 0.009±0.010^a^ | -0.066±0.03^a^ | -0.420±0.046^a^ |
|  |  |  | **Normal** | 0.308±0.026^b^ | 0.021±0.011^b^ | -0.106±0.03^b^ | -0.376±0.047^b^ |
|  |  |  | **Slow** | 0.266±0.027^c^ | 0.023±0.011^b^ | -0.136±0.03^c^ | -0.308±0.047^c^ |
| **Tibia** | **Knee** | **Rotational** | **Fast** | -0.018±0.031^a^ | -0.026±0.010^a^ | 0.031±0.009^a^ | -0.049±0.021^a^ |
|  |  |  | **Normal** | -0.004±0.034^a^ | -0.019±0.011^a,b^ | 0.016±0.011^a^ | -0.105±0.027^a^ |
|  |  |  | **Slow** | -0.019±0.036^a^ | -0.009±0.011^b^ | 0.034±0.012^a^ | -0.086±0.029^a^ |
|  |  | **Translational** | **Fast** | 0.366±0.025^a^ | 0.009±0.010^a^ | -0.066±0.03^a^ | -0.42±0.046^a^ |
|  |  |  | **Normal** | 0.308±0.026^b^ | 0.021±0.011^b^ | -0.106±0.03^b^ | -0.376±0.047^b^ |
|  |  |  | **Slow** | 0.266±0.027^c^ | 0.023±0.011^b^ | -0.136±0.03^c^ | -0.308±0.047^c^ |
|  | **Ankle** | **Rotational** | **Fast** | 0.119±0.018^a^ | 0.039±0.004^a^ | 0.168±0.024^a^ | 0.433±0.042^a^ |
|  |  |  | **Normal** | 0.079±0.022^a^ | 0.038±0.005^a^ | 0.188±0.025^a^ | 0.334±0.047^b^ |
|  |  |  | **Slow** | 0.078±0.024^a^ | 0.039±0.005^a^ | 0.233±0.025^b^ | 0.332±0.049^b^ |
|  |  | **Translational** | **Fast** | -0.079±0.012^a^ | 0.018±0.003^a^ | 0.151±0.011^a^ | 0.421±0.028^a^ |
|  |  |  | **Normal** | -0.060±0.012^b^ | 0.012±0.003^b^ | 0.153±0.012^a^ | 0.399±0.031^a^ |
|  |  |  | **Slow** | -0.050±0.012^b^ | 0.009±0.003^b^ | 0.148±0.012^a^ | 0.411±0.033^a^ |
| **Foot** | **Ankle** | **Rotational** | **Fast** | 0.066±0.012^a^ | 0.003±0.002^a^ | 0.135±0.010^a^ | 0.813±0.054^a^ |
|  |  |  | **Normal** | 0.053±0.015^a^ | 0.003±0.003^a^ | 0.130±0.012^a^ | 0.635±0.068^b^ |
|  |  |  | **Slow** | 0.052±0.016^a^ | 0.005±0.003^a^ | 0.160±0.013^a^ | 0.640±0.072^b^ |
|  |  | **Translational** | **Fast** | -0.079±0.012^a^ | 0.018±0.003^a^ | 0.151±0.011^a^ | 0.421±0.028^a^ |
|  |  |  | **Normal** | -0.060±0.012^b^ | 0.012±0.003^b^ | 0.153±0.012^a^ | 0.399±0.031^a^ |
|  |  |  | **Slow** | -0.050±0.012^b^ | 0.009±0.003^b^ | 0.148±0.012^a^ | 0.411±0.033^a^ |
|  | **Metatarsal** | **Rotational** | **Fast** | -0.000663±0.000062^a^ | -0.000001±0.000004^a^ | -0.000088±0.000006^a^ | -0.001496±0.000077^a^ |
|  |  |  | **Normal** | -0.000579±0.000063^b^ | -0.000006±0.000005^a^ | -0.000090±0.000007^a^ | -0.001333±0.000081^b^ |
|  |  |  | **Slow** | -0.000512±0.000064^c^ | -0.000008±0.000005^a^ | -0.000094±0.000007^a^ | -0.001182±0.000083^c^ |
|  |  | **Translational** | **Fast** | -0.001981±0.000988^a^ | 0.000010±0.000006^a^ | -0.000023±0.000008^a^ | 0.004±0.001^a^ |
|  |  |  | **Normal** | -0.000994±0.001001^b^ | 0.000008±0.000007^a^ | -0.000026±0.000008^a^ | 0.002±0.001^a^ |
|  |  |  | **Slow** | -0.000003±0.001010^c^ | 0.000015±0.000007^a^ | -0.000018±0.000008^a^ | 0.002±0.001^a^ |
| **Toe** | **Metatarsal** | **Rotational** | **Fast** | -0.000829±0.000093^a^ | -0.000007±0.000006^a^ | -0.000023±0.000010^a^ | -0.001864±0.000144^a^ |
|  |  |  | **Normal** | -0.000774±0.000094^a^ | -0.000003±0.000006^b^ | -0.000017±0.000010^a^ | -0.001614±0.000150^b^ |
|  |  |  | **Slow** | -0.000662±0.000095^b^ | -0.000006±0.000007^a^ | -0.000018±0.000010^a^ | -0.001325±0.000154^c^ |
|  |  | **Translational** | **Fast** | -0.001981±0.000988^a^ | 0.000010±0.000006^a^ | -0.000023±0.000008^a^ | 0.004±0.001^a^ |
|  |  |  | **Normal** | -0.000994±0.001001^b^ | 0.000008±0.000007^a^ | -0.000026±0.000008^a^ | 0.002±0.001^a^ |
|  |  |  | **Slow** | -0.000003±0.001010^c^ | 0.000015±0.000007^a^ | -0.000018±0.000008^a^ | 0.002±0.001^a^ |

Data are mean ± s.d. for all the trials across all the subjects. Different letters mean that the variable in a column differs significantly with each other (*p* < 0.05).
